# Association between vaccination status and COVID-19-related health outcomes among community-dwelling COVID-19 patients in Nara, Japan

**DOI:** 10.1265/ehpm.22-00199

**Published:** 2023-01-21

**Authors:** Kimiko Tomioka, Kenji Uno, Masahiro Yamada

**Affiliations:** 1 Nara Prefectural Health Research Center, Nara Medical University, Nara, Japan; 2 Chuwa Public Health Center of Nara Prefectural Government, Nara, Japan; 3 Department of Infectious Diseases, Minami-Nara General Medical Center, Nara, Japan

**Keywords:** COVID-19, Vaccination, COVID-19-related health outcomes, Omicron variant, Community-based study, Japan

## Abstract

**Background:**

Many previous studies have reported COVID-19 vaccine effectiveness, but there are few studies in Japan. This community-based, retrospective observational study investigated the association between vaccination status and COVID-19-related health outcomes in COVID-19 patients by SARS-CoV-2 variant type.

**Methods:**

The study participants were 24,314 COVID-19 patients aged 12 or older whose diagnoses were reported to the Nara Prefecture Chuwa Public Health Center from April 2021 to March 2022, during periods when the alpha, delta, and omicron variants of COVID-19 were predominant. The outcome variables were severe health consequences (SHC) (i.e., ICU admission and COVID-19-related death), hospitalization, and extension of recovery period. The explanatory variable was vaccination status at least 14 days prior to infection. Covariates included gender, age, population size, the number of risk factors for aggravation, and the number of symptoms at diagnosis. The generalized estimating equations of the multivariable Poisson regression models were used to estimate the adjusted incidence proportion (AIP) and 95% confidence interval (CI) for each health outcome. We performed stratified analyses by SARS-CoV-2 variant type, but the association between vaccination status and COVID-19-related health outcomes was stratified only for the delta and omicron variants due to the small number of vaccinated patients during the alpha variant.

**Results:**

Of the 24,314 participants, 255 (1.0%) had SHC; of the 24,059 participants without SHC, 2,102 (8.7%) were hospitalized; and of the 19,603 participants without SHC, hospitalization, and missing data on recovery period, 2,960 (15.1%) had extension of recovery period. Multivariable Poisson regression models showed that regardless of SARS-CoV-2 variant type or health outcome, those who received two or more vaccine doses had significantly lower risk of health outcomes than those who did not receive the vaccine, and there was a dose-response relationship in which the AIP for health outcomes decreased with an increased number of vaccinations.

**Conclusion:**

A higher number of vaccinations were associated with lower risk of COVID-19-related health outcomes, not only in the delta variant but also in the omicron variant. Our findings suggest that increasing the number of COVID-19 vaccine doses can prevent severe disease and lead to early recovery of patients not requiring hospitalization.

**Supplementary information:**

The online version contains supplementary material available at https://doi.org/10.1265/ehpm.22-00199.

## Graphical Abstract

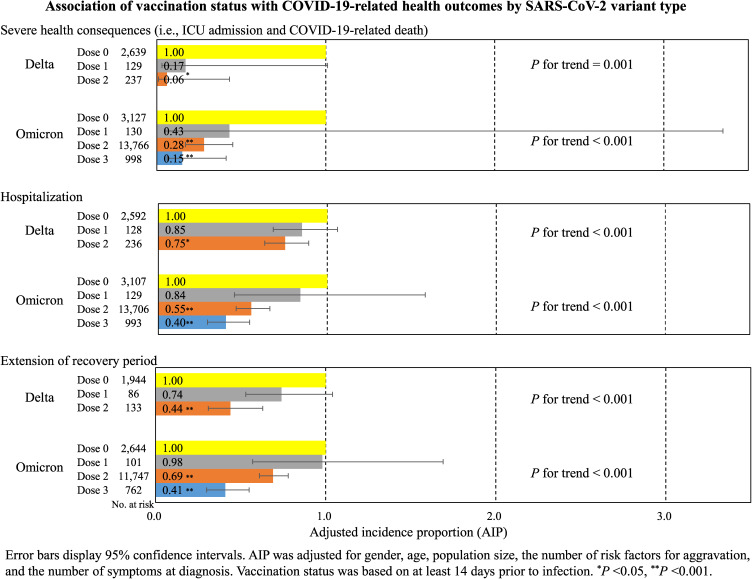

## Background

The Coronavirus disease 2019 (COVID-19) Omicron variant sub-lineages have progressed from the BA.2 lineage to the BA.4 and BA.5 lineages [1], and since July 2022, Japan has entered the 7th wave of COVID-19. The World Health Organization reported that the number of new weekly cases in Japan for the week of 1 to 7 August 2022 was 1,496,968, the highest number in the world for three consecutive weeks [2]. In Japan, it is an urgent issue to prevent the onset and reduce the severity of COVID-19 through vaccination as a countermeasure against the spread of infection.

A considerable number of randomized clinical trials have confirmed the safety and efficacy of COVID-19 vaccines [3–5], and many observational cohort studies and test-negative case-control studies on vaccine effectiveness have reported a significant reduction in infection, hospitalization, and progression to severe disease including death [6–10]. However, these previous studies have been Western-centric, and the number of studies from Asia, especially Japan, is small. Moreover, previous studies in Japan have been hospital-based, targeting healthcare professionals [11, 12]. Because healthcare workers are a young and energetic group, there is a limitation to the generalization of these results to members of the community. Therefore, it is necessary to examine the relationship between vaccination status and health outcomes in community-dwelling people in Japan. Furthermore, because COVID-19 infectivity has increased with the emergence of a new variant which can evade the vaccine-induced immune response [10, 13], the association between vaccination status and health outcomes should be evaluated for each variant type of the severe acute respiratory syndrome coronavirus 2 (SARS-CoV-2).

This study examined the association of COVID-19 vaccination status with three health outcomes (i.e., severe health consequences, hospitalization, and extension of recovery period) in community-dwelling COVID-19 patients by SARS-CoV-2 variant type, controlled for those factors identified in previous studies as risk factors for severe COVID-19, such as age, gender, comorbidities, and symptoms at diagnosis [14–17].

## Methods

### Study participants and data source

The study design is a community-based retrospective observational study. The potential study subjects were 30,495 persons whose diagnoses were reported by doctors to the Chuwa Public Health Center (PHC) as COVID-19, in accordance with the Infectious Diseases Control Law from 1 April 2021 to 31 March 2022. Because the target age for COVID-19 vaccination was 12 years or older during the period selected for this study, the study participants were limited to 24,314 persons aged 12 years or older. The details of the study population are provided in Additional file 1. We used data from the Health Center Real-time Information-sharing System on COVID-19 (HER-SYS) [18] related to COVID-19 infection notifications in accordance with the Infectious Diseases Control Law. The details of the HER-SYS are given in Additional file 2.

### The time of spread of each SARS-CoV-2 variant type

Of the six COVID-19 pandemic waves in Japan, the first to third waves between 1 April 2020 and 31 March 2021 were caused by the wild type, the fourth was caused by the alpha variant, the fifth by the delta variant, and the sixth by the BA.1 and BA.2 omicron variants. Since HER-SYS information was only available at the Chuwa PHC after 1 April 2021, the wild-type period could not be included as the target period in this study. Regarding the time of spread of each SARS-CoV-2 variant type, although there is no precise definition in Japan, we referred to the time periods used in previous studies [19–22] and the period of issuance of the state of emergency in Japan. Therefore, in this study, the period of the alpha variant was defined as being from 1 April 2021 to the end of June 2021, the period of the delta variant was from 1 July 2021 to the end of December 2021, and the period of BA.1 and BA.2 lineages of Omicron (hereinafter referred to as the omicron variant) was from 1 January 2022 to the end of March 2022.

### Health outcomes

This study included all laboratory-confirmed COVID-19 patients between 1 April 2021 and 31 March 2022 in an area under the jurisdiction of one PHC in Japan. Although we cannot assess the risk of COVID-19 infection, this study includes patients who did not require hospitalization (i.e., patients recovering at home or a lodging facility). Therefore, we added not only severe health consequences and hospitalization, which are important health outcomes to assess the efficacy and effectiveness of a vaccine [3–7, 9, 10], but also extension of recovery period. For each patient, we determined the presence or absence of each health outcome from the date of the COVID-19 diagnosis to the end of the isolation period.

#### Severe health consequences

According to the severity classification of the COVID-19 medical guidelines in Japan [23], severe cases are defined as those who have entered the intensive care unit (ICU) or who need a ventilator. In this study, persons with severe health consequences were defined as patients with ICU admission or COVID-19-related death.

#### Hospitalization

Cases who were hospitalized between the time they were diagnosed with COVID-19 and the time they were released from the isolation period were labeled as those with hospitalization. In this analysis, those who had severe health consequences were excluded from the analysis.

#### Extension of recovery period

According to Japanese standards during the selected period of this study [24], the isolation period for a COVID-19 patient was at least 10 days from the day after onset, and quarantine had to end at least 72 hours after the symptoms disappeared. Those who required an isolation period of 11 days or longer, which exceeded the national standard [24], were considered to have an extended recovery period. This analysis excluded those with missing data on recovery period, and included those who had neither severe health consequences nor hospitalization (i.e., patients recovering at home or a lodging facility).

### Explanatory variable

The explanatory variable was vaccination status. The vaccines used in most of the vaccinated population in the study area during the study period were BNT16b2 (Pfizer/BioNtech) and mRNA-1273 (Moderna). Although it takes about 2 weeks from vaccination to develop immunity [4, 25], the BNT16b2 vaccine has been confirmed to be effective ≥7 days after the second and booster doses [4, 26, 27]. Therefore, first, we evaluated the number of vaccinations at least 14 days prior to infection. Next, an additional analysis was performed by recalculating the number of vaccinations at least 14 days after the first dose and at least 7 days after the second and third doses. The SARS-CoV-2 variant type of vaccination status was categorized based on each patient’s date of onset. The details of vaccination status in each COVID-19 variant period are described in Additional file 3.

COVID-19 patients without data on vaccination status were more likely to develop severe health outcomes or had a longer recovery period than those with data on vaccination status (Additional file 4). This suggests that excluding persons with unknown vaccination status from the analysis may cause selection bias. Therefore, in this study, those who had missing data on vaccination status were designated in the analysis as missing [28]. When confirming a dose-response relationship between an increased number of vaccinations and a decreased risk of an outcome variable, the missing group was excluded from the analysis.

### Covariates

Based on previous studies [14–17], the following variables were adopted as covariates which were potential confounding factors in the association between vaccination status and health outcomes: gender, age, population size, the number of risk factors for aggravation, and the number of symptoms at diagnosis. To deal with missing covariates, we conducted multiple imputations by chained equations implemented in IBM SPSS Missing Value Version 27. The details of the covariates and multiple imputations are described in Additional file 5, and information including the number of missing values according to the SARS-CoV-2 variant type is shown in Additional file 6. For the issue of multicollinearity, we confirmed that no variable had a variance inflation factor value greater than 2.

### Statistical analysis

Data comparisons of proportions between groups were tested using the chi-squared test. The Cochran-Armitage test was used to perform a trend test to detect the decreased or increased prevalence or cumulative incidence.

To investigate the association between vaccination status and health outcome, we used the generalized estimating equations of the multivariable Poisson regression models after simultaneously adjusting for all covariates to calculate the adjusted incidence proportion (AIP) with 95% confidence interval (CI) for severe health consequences, hospitalization, or extension of the recovery period. An independent variable was the vaccination status, with the unvaccinated group as the reference.

Although we performed a stratified analysis by SARS-CoV-2 variant type, the association between vaccination status and health outcome was evaluated for the delta variant and the omicron variant only, because of the small number of vaccinated individuals during the alpha variant. Statistical analyses were performed using the IBM SPSS Statistics Ver. 27 for Windows (Armonk, New York, US), and a significant level was set at 0.05 (two-tailed test).

### Ethical issues/ethical considerations

This study was approved by the Nara Medical University Ethics Committee (Approval No. 3262). Informed consent was not required because data collection was in accordance with the Infectious Diseases Control Law. However, when performing data analysis for research, we used data with personal information removed, and published an opt-out document on the Chuwa PHC website to ensure that patients had the opportunity to withdraw.

## Results

The mean observation period was 10.8 days (standard deviation, 5.3). For the cumulative incidence during the observation period, of the 24,314 participants, 255 individuals (1.0%) had severe health consequences. Of the 24,059 participants without severe health consequences, 2,102 individuals (8.7%) had hospitalization. Of the 19,603 participants without severe health consequences, hospitalization, and missing data on recovery period, 2,960 individuals (15.1%) had extension of recovery period. Among the 24,314 participants, the mean age was 41.8 (standard deviation, 20.0), the proportion of people aged 65 and older was 14.9%, and the proportion of men was 48.5%. For vaccination status, 7,379 participants (30.3%) were unvaccinated, 263 (1.1%) received one dose of vaccine, 14,004 (57.6%) received two vaccine doses, 998 (4.1%) received a third vaccine dose, and 1,670 (6.9%) were of unknown vaccination status.

**Fig. 1:**
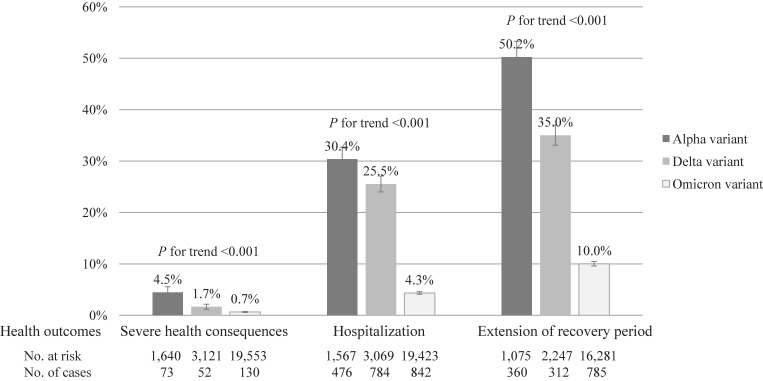
Cumulative incidence of individuals with a health outcome by SARS-CoV-2 variant type. A trend test was performed to detect a decrease in the cumulative incidence of a health outcome with the emergence of new variants using the Cochran-Armitage test.

Regarding characteristics of the study participants by SARS-CoV-2 variant type (left side of Table 1), during the alpha variant, older people, those with aggravation risk factors, and the unvaccinated were more common. The delta variant period had the lowest proportion of older people. In the omicron variant, there were few men and many vaccinated people. Regarding characteristics by health outcome (right side of Table 1), the more severe participant health outcomes were, the more frequently they were men, older, people with aggravation risk factors, people with symptoms at diagnosis, and the unvaccinated. Population size was not associated with the level of severity of health outcomes. Regarding the cumulative incidence of individuals with a health outcome by SARS-CoV-2 variant type (Fig. 1), all health outcomes were highest in the period of the alpha variant, and the cumulative incidence tended to decrease significantly as new variants emerged (*P* for trend <0.001 in all outcomes).

**Table 1:** Characteristics of the study participants by SARS-CoV-2 variant type or by health outcomes

	**Entire period**	**SARS-CoV-2 variant type**			***P* ^a^**	**Health outcomes**				***P* ^b^**
		**Alpha**	**Delta**	**Omicron**		**None**	**Extension of recovery period**	**Hospitalization**	**Severe health consequences**	
	**n = 24,314**	**n = 1,640**	**n = 3,121**	**n = 19,553**		**n = 20,500**	**n = 1,457**	**n = 2,102**	**n = 255**	
Gender: men	48.5%	52.8%	53.1%	47.4%	<0.001	48.1%	47.6%	50.9%	61.6%	<0.001
Age: 65 years or older	14.9%	19.4%	6.4%	15.9%	<0.001	11.4%	15.0%	43.1%	63.9%	<0.001
Population size: ≥120,000	20.5%	21.3%	23.0%	20.0%	<0.001	20.4%	19.7%	22.3%	16.2%	0.437
No. of aggravation risk factors: ≥1	27.7%	39.3%	34.5%	25.7%	<0.001	24.0%	29.1%	57.7%	74.0%	<0.001
No. of symptoms at diagnosis: ≥3	20.8%	15.1%	22.5%	21.0%	<0.001	20.5%	20.2%	23.5%	24.9%	0.001
Vaccination status: unvaccinated	30.3%	98.4%	84.6%	16.0%	<0.001	25.6%	55.5%	56.7%	54.9%	<0.001

^a^Chi-squared test. ^b^Cochran-Armitage test.

AIPs for severe health consequences are presented in Additional file 7A. For all SARS-CoV-2 variant types, older age was a strong predictor of severe health consequences. The risk of severe health consequences was significantly lower in females than in males for the alpha and omicron variants. AIPs for hospitalization are presented in Additional file 7B. For all SARS-CoV-2 variant types, the risk of hospitalization was significantly higher in older people, those with more risk factors for aggravation, and those with more symptoms at diagnosis. AIPs for extension of recovery period are shown in Additional file 7C. Aggravation risk factors and symptoms at diagnosis were not associated with the extension of recovery period for any of the SARS-CoV-2 variant types. Unlike the foregoing health outcomes, older age and male gender were not a strong predictor of extension of recovery period.

**Fig. 2:**
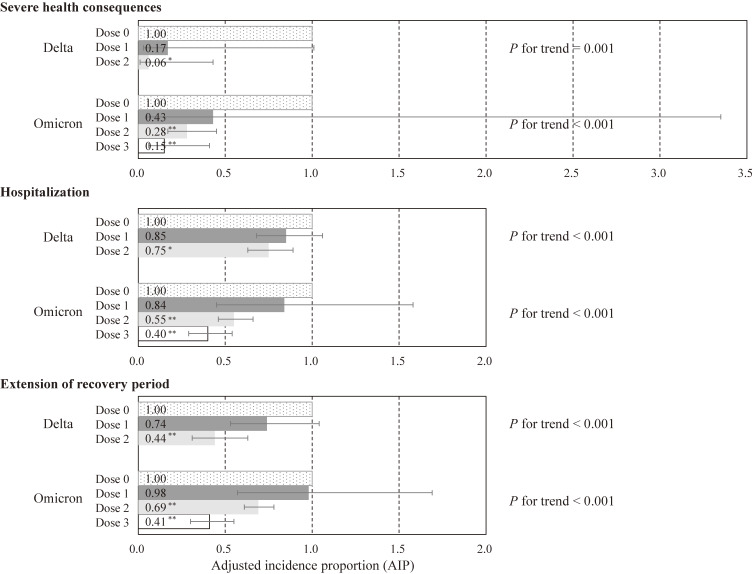
Association of vaccination status with COVID-19-related health outcomes by SARS-CoV-2 variant type. Error bars display 95% confidence intervals. AIP was adjusted for gender, age, population size, the number of risk factors for aggravation, and the number of symptoms at diagnosis. Vaccination status was based on at least 14 days prior to infection. *<0.050, **<0.001.

In terms of the association of vaccination status with health outcomes (Fig. 2), regardless of SARS-CoV-2 variant type or health outcome, those who received two or more vaccine doses had significantly lower risk of health outcomes than those who did not receive the vaccine, no significant difference was found between patients who received one dose of vaccine and unvaccinated patients, and there was a dose-response relationship in which the AIP for health outcomes decreased with an increased number of vaccinations. Compared to unvaccinated individuals, two vaccine doses in the delta variant (AIP, 0.06; 95% CI, 0.01–0.43), two vaccine doses in the omicron variant (AIP, 0.28; 95% CI, 0.17–0.45), and three vaccine doses in the omicron variant (AIP, 0.15; 95% CI, 0.06–0.41) were associated with a lower risk of severe health consequences; two vaccine doses in the delta variant (AIP, 0.75; 95% CI, 0.63–0.89), two vaccine doses in the omicron variant (AIP, 0.55; 95% CI, 0.46–0.66), and three vaccine doses in the omicron variant (AIP, 0.40; 95% CI, 0.29–0.54) were associated with a lower risk of hospitalization; and two vaccine doses in the delta variant (AIP, 0.44; 95% CI, 0.31–0.63), two vaccine doses in the omicron variant (AIP, 0.69; 95% CI, 0.61–0.78), and three vaccine doses in the omicron variant (AIP, 0.41; 95% CI, 0.30–0.55) were associated with a lower risk of extension of recovery period.

Regarding effects on health outcomes in those with unknown vaccination status, AIP for extension of recovery period was significantly lower in the delta variant, and higher in the omicron variant, suggesting that it may be underestimated or overestimated due to the influence of persons with unknown vaccination status (Additional files 7C).

Additional analyses of the number of vaccinations at least 14 days after the first dose and at least 7 days after the second and third doses showed that AIP for hospitalization during the delta variant was significantly different between 1st and no vaccinations, but other than that, the results showed the same tendency as the results for vaccination status at least 14 days prior to infection (Additional files 8A–8C).

## Discussion

Using community-based data, this study showed that more COVID-19 vaccinations were associated with a lower risk for COVID-19-related health outcomes among COVID-19 patients. To our knowledge, this study is the first report to examine the association between COVID-19 vaccination status and health outcomes in members of the community in Japan.

The number of infected people with COVID-19 has exploded with the appearance of new SARS-CoV-2 variant types, which suggests that the COVID-19 vaccine cannot be expected to have a sufficient infection-preventing effect against new variants of COVID-19 [6, 10, 13]. The results of this study showed that for the omicron variant, the risk of severe health consequences decreased as the number of vaccinations increased, and there was no significant difference between the unvaccinated and individuals with a single vaccination. These results suggest that complete vaccination and booster vaccination are effective in preventing the aggravation of COVID-19 for a new SARS-CoV-2 variant type. Furthermore, this study found that 2 or more doses of vaccine for both the delta and omicron variants can prevent not only severe health consequences and hospitalization, but also the extension of recovery period in patients recovering at home or a lodging facility.

Variant-specific association between vaccination status and COVID-19-related health outcomes was previously studied in the US [29] and Qatar [30]. A cohort study of 10.6 million residents of North Carolina in the US [29] reported that those with two doses of the COVID-19 vaccines compared with one dose and those with booster vaccination compared with two doses were significantly associated with lower risk of SARS-CoV-2 infection and severe COVID-19 outcomes (i.e., COVID-19-related hospitalization and death). Furthermore, the US study [29] classified the 15-month study period into pre-delta, delta, and omicron by variant type, and reported that the preventive effect of vaccination against infection was diminished by the emergence of new variant types, but the vaccine effectiveness against severe COVID-19 outcomes remained high during the omicron variant. A cohort study of 2.2 million persons who had received at least two doses of COVID-19 vaccines in Qatar [30] also found that a booster vaccination, compared with the two-dose primary series, was less effective against infection for the omicron variant than for the delta variant, but had a strong protection against COVID-19-related hospitalization and death caused by both the omicron and delta variants. These two [29, 30] were community-based studies on the effectiveness of booster vaccination against the risk of infection and severe outcomes. Although these previous studies had a different research purpose from our study, they suggest that COVID-19 vaccines increase the protective effect on severe COVID-19 outcomes as the number of doses increases, and that the protection is maintained even for the omicron variant.

This study has several strengths. First, we assessed the risk of health outcomes for all infected individuals in a region. Second, we have shown that the COVID-19 vaccine is effective against both the delta and omicron variants in reducing three health outcomes, even after adjusting for important risk factors of COVID-19 aggravation such as gender, age, comorbidities, smoking, and obesity [14–16].

This study has several limitations. First, the HER-SYS information has more errors and missing values than information from active epidemiological investigation [31]. Those with health outcomes (especially severe health consequences) had more missing data than those without health outcomes (Additional file 4), and those with unknown vaccination status had significantly fewer people with extension of recovery period during the delta variant and significantly more during the omicron variant than those who were unvaccinated (Additional file 7C). If those with health outcomes selectively include unvaccinated individuals, it may result in an underestimation in the association between vaccination status and health outcomes. Second, although our study used vaccination status as an explanatory variable, we failed to reflect the type of vaccine. Regarding 1 and 2 doses of the COVID-19 vaccines in Japan, the total number of vaccinations by vaccine type has been published [32]. Based on this data as of 19 August 2022, out of a total of 206,616,825 doses, the Pfizer/BioNTech COVID-19 vaccine had 84.1% and the Moderna COVID-19 vaccine had 15.8%, these two accounting for 99.9% of total vaccines administered. Therefore, the vaccine administered in Japan was an mRNA-based vaccine, and this study evaluated the association of mRNA-based vaccine with health outcomes. Future studies should evaluate the vaccine-health association of each vaccine type in Japanese people, including the omicron variant BA.5 and emerging variants. Third, regarding comorbidities at risk of aggravation, HER-SYS data did not include coronary heart disease and cerebrovascular disease during the study period. Because both coronary heart disease and cerebrovascular disease are important risk factors for the severity of COVID-19 [14–17], future studies should verify the vaccine-health association including these diseases. Finally, this study targeted a PHC in Nara Prefecture. Although the COVID-19 infection status and vaccination status in the target PHC are roughly the national average [33, 34], generalization of the results obtained to the whole country requires a cautious attitude.

Despite the above limitations, this study is valuable as the first study in Japan to verify the association between vaccination status and health outcomes for all infected people in the community, including patients recovering at home/a lodging facility. Based on our findings, policy makers should promote measures to increase the number of vaccinations to prevent severe disease and hospitalization from COVID-19 (that is, to prevent hospitals from being overwhelmed by COVID-19). Family physicians should encourage patients to receive the full vaccination and booster dose program for early recovery from COVID-19.

In conclusion, we investigated the association between vaccination status and health outcomes using data from all COVID-19 patients from April 2021 to March 2022 at a PHC in Japan. The results of this study showed that a dose-response relationship between a higher number of vaccinations and lower risk of COVID-19-related health outcomes was observed not only during the delta period, but also during the omicron period. Although the number of people infected with COVID-19 has been increasing due to the emergence of a new variant, our findings suggest that increasing the number of doses of COVID-19 vaccines can prevent severe disease and hospitalization caused by the omicron variant, and that it can lead to early recovery of patients not requiring hospitalization.

## Abbreviations

AIP: adjusted incidence proportion
CI: confidence interval
COVID-19: Coronavirus disease 2019
HER-SYS: Health Center Real-time Information-sharing System on COVID-19
ICU: intensive care unit
PHC: public health center
SARS-CoV-2: severe acute respiratory syndrome coronavirus 2

## Supplementary information

**Additional file 1.** Details of study population. **Additional file 2.** Details of the Health Center Real-time Information-sharing System on COVID-19 (HER-SYS). **Additional file 3.** Details of vaccination status in each COVID-19 variant period. **Additional file 4.** Characteristics of the study participants by vaccination status. **Additional file 5.** Detailed explanations of covariates and multiple imputations. **Additional file 6.** Characteristics of COVID-19 patients ≥12 years of age, by SARS-CoV-2 variant type. **Additional file 7A.** AIP for severe health consequences by SARS-CoV-2 variant type: COVID-19 patients aged 12 and over. **Additional file 7B.** AIP for hospitalization by SARS-CoV-2 variant type: COVID-19 patients aged ≥12 without severe health consequences. **Additional file 7C.** AIP for extension of recovery period by SARS-CoV-2 variant type: COVID-19 patients not requiring hospitalization. **Additional file 8.** Vaccination status ≥14 days after the first-dose and ≥7 days after the 2–3 doses. **8A.** AIP for severe health consequences by SARS-CoV-2 variant type. **8B.** AIP for hospitalization by SARS-CoV-2 variant type. **8C.** AIP for extension of recovery period by SARS-CoV-2 variant type.

## Declarations

### Availability of data and materials

The data analyzed in this study are not publicly available.
