# Supplementary material for: Association between vaccination status and COVID-19-related health outcomes among community-dwelling COVID-19 patients in Nara, Japan
Source: Environ Health Prev Med. 2023 Jan 21;28:7. doi: 10.1265/ehpm.22-00199 (PMC9884564; doi:10.1265/ehpm.22-00199)
Supplement: Supplementary file 1 — Additional file 1. Details of study population. Additional file 2. Details of the Health Center Real-time Information-sharing System on COVID-19 (HER-SYS). Additional file 3. Details of vaccination status in each COVID-19 variant period. Additional file 4. Characteristics of the study participants by vaccination status. Additional file 5. Detailed explanations of covariates and multiple imputations. Additional file 6. Characteristics of COVID-19 patients ≥12 years of age, by SARS-CoV-2 variant type. Additional file 7A. AIP for severe health consequences by SARS-CoV-2 variant type: COVID-19 patients aged 12 and over. Additional file 7B. AIP for hospitalization by SARS-CoV-2 variant type: COVID-19 patients aged ≥12 without severe health consequences. Additional file 7C. AIP for extension of recovery period by SARS-CoV-2 variant type: COVID-19 patients not requiring hospitalization. Additional file 8. Vaccination status ≥14 days after the first-dose and ≥7 days after the 2–3 doses. 8A. AIP for severe health consequences by SARS-CoV-2 variant type. 8B. AIP for hospitalization by SARS-CoV-2 variant type. 8C. AIP for extension of recovery period by SARS-CoV-2 variant type. [file ehpm-28-007-s001.docx]

**Additional file 1** Details of study population

The target area for this study was the jurisdiction of the Chuwa PHC of the Nara Prefectural Government in Japan, which includes 18 municipalities (7 cities, 8 towns, and 3 villages) in the northern part of Nara Prefecture. The municipalities under its jurisdiction range from a medium-sized city near Osaka, where the population is still increasing, to a depopulated village with a population of less than 1,500. According to the 2021 estimated population [1], the population of municipalities under the jurisdiction of Chuwa PHC accounts for 42.4% of the population of Nara Prefecture.

Because COVID-19 infection status and the vaccination coverage were aggregated and published by prefecture, only data for Nara prefecture was available. As of 12 August 2022, Nara Prefecture rated 20th out of 47 prefectures in terms of the number of new weekly cases per 100,000 population in the last week [2], and third vaccination coverage was 63.0%, which was about the same as the national average (63.6%) [3].

References

1. Statistical Analysis Division, Governor's Office, General Affairs Department, Nara Prefecture. Nara Prefecture Estimated Population Annual Report. March 2022. https://www.pref.nara.jp/6265.htm. Accessed 20 Aug 2022. (Japanese).
2. Ministry of Health, Labour and Welfare, Japan. Visualizing the data: information on COVID-19 infections. https://covid19.mhlw.go.jp/. Accessed 20 Aug 2022.
3. Digital Agency. Vaccination status of the COVID-19 vaccine. https://info.vrs.digital.go.jp/dashboard/. Accessed 20 Aug 2022. (Japanese).

**Additional file 2** Details of the Health Center Real-time Information-sharing System on COVID-19 (HER-SYS)

The HER-SYS was developed and operated by the Ministry of Health, Labour and Welfare. By utilizing this system, patients recovering at home can easily report their daily health status on their smartphones, and inpatient information such as test results and intensive care unit admission can be continuously recorded, thereby reducing the workload of PHC and streamlining information sharing and management [1]. On the other hand, because the HER-SYS information is entered by the doctor (medical institution) who diagnosed the COVID-19 patient, it is less accurate [2] than the information from active epidemiological investigation [3], in which public health nurses working at PHC call each patient to collect information. The system of active epidemiological investigation was unable to cope with the increase in the number of patients after the 6th wave caused by the spread of Omicron BA.1 and BA.2 (i.e., after January 2022), and in the absence of information on the duration of the omicron variant. To evaluate the effect of vaccination status on SARS-CoV-2 variant types including the omicron variant, we used HER-SYS information.

References

1. Ministry of Health, Labour and Welfare, Japan. Health Center Real-time Information-sharing System on COVID-19 (HER-SYS). https://www.mhlw.go.jp/stf/seisakunitsuite/bunya/0000121431_00129.html. Accessed 20 Aug 2022. (Japanese).
2. Ministry of Health, Labour and Welfare, Japan. Efforts to improve accuracy control of HER-SYS input data. (Administrative contact) November 16, 2020. https://www.mhlw.go.jp/content/10900000/000695261.pdf. Accessed 20 Aug 2022. (Japanese).
3. National Institute of Infectious Diseases. Manual for active epidemiological surveillance of patients with novel coronavirus infection (provisional version). https://www.niid.go.jp/niid/en/2019-ncov-e/2484-idsc/9472-2019-ncov-02-en.html. Accessed 20 Aug 2022.

**Additional file 3** Details of vaccination status in each COVID-19 variant period

The period of the alpha variant is the period when healthcare workers and older people in Japan had priority access to vaccination [1,2], and the first vaccination coverage excluding healthcare workers was 1.4% as of 15 May 2021, and 23.1% as of 30 June 2021 [3]. Due to the small number of vaccinated individuals during the alpha variant period (i.e., 4 people for the first vaccination and one person for the second vaccination), we abandoned assessing the association between vaccination status and health outcomes during the alpha variant period.

The third vaccination started on 1 December 2021, and the third vaccination coverage including healthcare workers was 0.82% as of 31 December 2021 [3]. Since no one in this study received a third dose of the COVID-19 vaccine during the delta variant period, vaccination status during the delta period was classified into unvaccinated, dose 1, or dose 2.

References

1. Naito T, Yan Y, Tabe Y, Seyama K, Deshpande GA. Real-world evidence for the effectiveness and breakthrough of BNT162b2 mRNA COVID-19 vaccine at a medical center in Japan. Hum Vaccin Immunother. 2022;1:1-2.
2. Nomoto H, Hayakawa K, Ohmagari N. Impact of prioritized vaccinations for the elderly on the COVID-19 pandemic in Japan. Glob Health Med. 2022;4:129-32.
3. Digital Agency. Vaccination status of the COVID-19 vaccine. https://info.vrs.digital.go.jp/dashboard/. Accessed 20 Aug 2022. (Japanese).

**Additional file 4** Characteristics of the study participants by vaccination status

|  | Vaccination status | | *P^a^* |
| --- | --- | --- | --- |
|  | Identified | Missing |  |
|  | n = 22,644 | n = 1,670 |  |
| Gender: men | 48.3% | 50.3% | 0.122 |
| Age: 65 years or older | 14.2% | 24.5% | <0.001 |
| Missing for population size | 7.1% | 15.6% | <0.001 |
| Missing for risk factors for aggravation | 18.7% | 50.7% | <0.001 |
| Missing for symptoms at diagnosis | 3.2% | 3.7% | 0.314 |
| Patients who had severe health consequences | 0.9% | 2.8% | <0.001 |
|  | n = 22,436 | n = 1,623 |  |
| Patients with hospitalization^b^ | 8.7% | 9.6% | 0.202 |
|  | n = 18,411 | n = 1,113 |  |
| Patients with extension of recovery period^c^ | 7.3% | 10.1% | <0.001 |

^a^Chi-squared test.

^b^The analyzed subjects were COVID-19 patients ≥12 years of age without severe health consequences.

^c^The analyzed subjects were COVID-19 patients ≥12 years of age recovering at home or a lodging facility without missing data on recovery period.

**Additional file 5** Detailed explanations of covariates and multiple imputations

Covariates

Age was standardized (mean = 41.8 years; standard deviation = 20.0 years), and evaluated per 1 standard deviation increase. Municipalities of residence were classified into 4 groups according to population size: population of less than 30,000, population of 30,000 or more and less than 60,000, population of 60,000 or more and less than 120,000, and population of 120,000 or more. Population size was a discrete variable ranging from 1 to 4, where 1 represents the least populated (less than 30,000), and 4 represents the most populated (more than 120,000). In reference to previous studies [1-3], we selected nine risk factors for aggravation: chronic obstructive pulmonary disease, diabetes, chronic kidney disease, malignant tumors, hypertension, dyslipidemia, smoking, obesity with a body mass index of 30 or higher, and pregnancy. Of these nine, the total number of applicable risk factors was calculated for each patient. The number of risk factors for aggravation was a discrete variable ranging from 0 to 2, where 0 represents no risk factors, and 2 represents 2 or more risk factors. Based on a previous Japanese study [4], symptoms at diagnosis included fever, cough, acute respiratory symptoms other than cough, pneumonia, severe pneumonia, acute respiratory distress syndrome, and general malaise. Then, the total number of these 7 symptoms was calculated. The number of symptoms at diagnosis was a discrete variable ranging from 0 to 4, where 0 represents no symptoms at diagnosis, and 4 represents 4 or more symptoms at diagnosis.

Multiple imputations

To deal with missing covariates, we conducted multiple imputations by chained equations [5]. Using logistic regression, we created five sets of imputation data and performed analyses on the complete pooled data set. Gender, age, population size, the number of risk factors for aggravation, the number of symptoms at diagnosis, the variant type, vaccination status, and severe health consequences were entered into the imputation procedure. For this study, there were no missing data on gender and age. Population size (7.7%), the number of risk factors for aggravation (20.9%), and the number of symptoms at diagnosis (3.2%) were imputed as ordinal variables.

References

1. Bentivegna M, Hulme C, Ebell MH. Primary Care Relevant Risk Factors for Adverse Outcomes in Patients With COVID-19 Infection: A Systematic Review. J Am Board Fam Med. 2021;34(Suppl):S113-26.
2. Dorjee K, Kim H, Bonomo E, Dolma R. Prevalence and predictors of death and severe disease in patients hospitalized due to COVID-19: A comprehensive systematic review and meta-analysis of 77 studies and 38,000 patients. PLoS One. 2020;15:e0243191.
3. Figliozzi S, Masci PG, Ahmadi N, et al. Predictors of adverse prognosis in COVID-19: A systematic review and meta-analysis. Eur J Clin Invest. 2020;50:e13362.
4. amada G, Hayakawa K, Matsunaga N, et al. Predicting respiratory failure for COVID-19 patients in Japan: a simple clinical score for evaluating the need for hospitalisation. Epidemiology and Infection 2021;149:e175.
5. Azur MJ, Stuart EA, Frangakis C, Leaf PJ. Multiple imputation by chained equations: what is it and how does it work? Int J Methods Psychiatr Res. 2011;20:40-9.

**Additional file 6** Characteristics of COVID-19 patients ≥12 years of age, by SARS-CoV-2 variant type

|  |  | Entire period  (n = 24,314) | SARS-CoV-2 variant type | | | *P*^a^ |
| --- | --- | --- | --- | --- | --- | --- |
|  |  |  | Alpha  (n = 1,640) | Delta  (n = 3,121) | Omicron  (n = 19,553) |  |
|  |  | n (%) | n (%) | n (%) | n (%) |  |
| Gender | Male | 11,785 (48.5) | 866 (52.8) | 1,656 (53.1) | 9,263 (47.4) | <0.001 |
|  | Female | 12,529 (51.5) | 774 (47.2) | 1,465 (47.0) | 10,290 (52.6) |  |
| Age | Aged 12 to 40 | 12,658 (52.1) | 694 (42.3) | 1,828 (58.6) | 10,136 (51.8) | <0.001 |
|  | Aged 41 to 60 | 7,206 (29.6) | 565 (34.5) | 1,008 (32.3) | 5,633 (28.8) |  |
|  | Aged 61 to 74 | 2,541 (10.5) | 229 (14.0) | 189 (6.1) | 2,123 (10.9) |  |
|  | Aged 75 or older | 1,909 (7.9) | 152 (9.3) | 96 (3.1) | 1,661 (8.5) |  |
| Population  size | <30,000 | 5,442 (22.4) | 427 (26.0) | 589 (18.9) | 4,426 (22.6) | <0.001 |
|  | 30,000 to 59,999 | 6,329 (26.0) | 445 (27.1) | 819 (26.3) | 5,065 (25.9) |  |
|  | 60,000 to 119,999 | 6,056 (24.9) | 401 (24.5) | 968 (31.0) | 4,687 (24.0) |  |
|  | 120,000 or more | 4,616 (19.0) | 345 (21.0) | 713 (22.9) | 3,558 (18.2) |  |
|  | Missing | 1,871 (7.7) | 22 (1.3) | 32 (1.0) | 1,817 (9.3) |  |
| Number of  risk factors for aggravation | 0 | 13,979 (57.5) | 580 (35.4) | 1,183 (37.9) | 12,216 (62.5) | <0.001 |
|  | 1 | 3,955 (16.3) | 288 (17.6) | 499 (16.0) | 3,168 (16.2) |  |
|  | 2+ | 1,293 (5.3) | 96 (5.9) | 126 (4.0) | 1,071 (5.5) |  |
|  | Missing | 5,087 (20.9) | 676 (41.2) | 1,313 (42.1) | 3,098 (15.8) |  |
| Number of  symptoms  at diagnosis | 0 | 2,206 (9.1) | 357 (21.8) | 447 (14.3) | 1,402 (7.2) | <0.001 |
|  | 1 | 7,311 (30.1) | 442 (27.0) | 832 (26.7) | 6,037 (30.9) |  |
|  | 2 | 9,112 (37.5) | 531 (32.4) | 1,078 (34.6) | 7,503 (38.4) |  |
|  | 3+ | 4,904 (20.2) | 235 (14.3) | 685 (22.0) | 3,984 (20.4) |  |
|  | Missing | 781 (3.2) | 75 (4.6) | 79 (2.5) | 627 (3.2) |  |
| Vaccination  status | Unvaccinated | 7,379 (30.3) | 1,613 (98.4) | 2,639 (84.6) | 3,127 (16.0) | <0.001 |
|  | Dose 1 | 263 (1.1) | 4 (0.2) | 129 (4.1) | 130 (0.7) |  |
|  | Dose 2 | 14,004 (57.6) | 1 (0.1) | 237 (7.6) | 13,766 (70.4) |  |
|  | Dose 3 | 998 (4.1) | 0 (0.0) | 0 (0.0) | 998 (5.1) |  |
|  | Missing | 1,670 (6.9) | 22 (1.3) | 116 (3.7) | 1,532 (7.8) |  |

^a^Chi-squared test.

**Additional file 7A** AIP for severe health consequences by SARS-CoV-2 variant type: COVID-19 patients aged 12 and over

|  |  | Entire period (n = 24,314) | | |  | SARS-CoV-2 variant type | | | | | | | | | | |
| --- | --- | --- | --- | --- | --- | --- | --- | --- | --- | --- | --- | --- | --- | --- | --- | --- |
|  |  |  |  |  |  | Alpha (n = 1,640) | | |  | Delta (n = 3,121) | | |  | Omicron (n = 19,553) | | |
|  |  | n | AIP^a^ (95% CI） | *P* |  | n | AIP^b^ (95% CI） | *P* |  | n | AIP^c^ (95% CI） | *P* |  | n | AIP^c^ (95% CI） | *P* |
| Gender | Male | 11,785 | 1.00 |  |  | 866 | 1.00 |  |  | 1,656 | 1.00 |  |  | 9,263 | 1.00 |  |
|  | Female | 12,529 | 0.55 (0.43-0.70) | <0.001 |  | 774 | 0.43 (0.27-0.67) | <0.001 |  | 1,465 | 0.58 (0.32-1.04) | 0.069 |  | 10,290 | 0.60 (0.43-0.85) | 0.004 |
| Age^d^ (per 1 SD increment) | |  | 3.97 (3.44-4.60) | <0.001 |  |  | 2.79 (2.21-3.54) | <0.001 |  |  | 3.39 (2.54-4.53) | <0.001 |  |  | 5.29 (4.15-6.74) | <0.001 |
| Population size^e^ | |  | 0.97 (0.87-1.08) | 0.542 |  |  | 0.84 (0.68-1.04) | 0.102 |  |  | 1.26 (0.95-1.66) | 0.105 |  |  | 0.96 (0.83-1.12) | 0.612 |
| No. of risk factors for aggravation^f^ | |  | 1.54 (1.22-1.94) | 0.001 |  |  | 1.45 (1.05-2.01) | 0.023 |  |  | 1.42 (0.91-2.22) | 0.122 |  |  | 1.64 (1.18-2.27) | 0.005 |
| No. of symptoms at diagnosis^g^ | |  | 1.22 (1.07-1.39) | 0.004 |  |  | 1.23 (0.98-1.55) | 0.070 |  |  | 1.60 (1.19-2.15) | 0.002 |  |  | 1.11 (0.90-1.36) | 0.318 |
| Variant type | Alpha | 1,640 | 1.00 |  |  |  |  |  |  |  |  |  |  |  |  |  |
|  | Delta | 3,121 | 1.06 (0.73-1.53) | 0.760 |  |  |  |  |  |  |  |  |  |  |  |  |
|  | Omicron | 19,553 | 0.47 (0.32-0.68) | <0.001 |  |  |  |  |  |  |  |  |  |  |  |  |
| Vaccination status^h^ | Unvaccinated | 7,379 | 1.00 |  |  | 1,613 |  |  |  | 2,639 | 1.00 |  |  | 3,127 | 1.00 |  |
|  | Dose 1 | 263 | 0.22 (0.06-0.85) | 0.028 |  | 4 |  |  |  | 129 | 0.17 (0.03-1.01) | 0.051 |  | 130 | 0.43 (0.05-3.35) | 0.417 |
|  | Dose 2 | 14,004 | 0.23 (0.16-0.32) | <0.001 |  | 1 |  |  |  | 237 | 0.06 (0.01-0.43) | 0.005 |  | 13,766 | 0.28 (0.17-0.45) | <0.001 |
|  | Dose 3 | 998 | 0.15 (0.06-0.39) | <0.001 |  | 0 |  |  |  |  |  |  |  | 998 | 0.15 (0.06-0.41) | <0.001 |
|  | Unknown | 1,670 | 0.96 (0.64-1.46) | 0.859 |  | 22 |  |  |  | 116 | 1.37 (0.41-4.60) | 0.607 |  | 1,532 | 0.97 (0.57-1.64) | 0.904 |

**Additional file 7B** AIP for hospitalization by SARS-CoV-2 variant type: COVID-19 patients aged ≥12 without severe health consequences

|  |  | Entire period (n = 24,059) | | |  | SARS-CoV-2 variant type | | | | | | | | | | |
| --- | --- | --- | --- | --- | --- | --- | --- | --- | --- | --- | --- | --- | --- | --- | --- | --- |
|  |  |  |  |  |  | Alpha (n = 1,567) | | |  | Delta (n = 3,069) | | |  | Omicron (n = 19,423) | | |
|  |  | n | AIP^a^ (95% CI） | *P* |  | n | AIP^b^ (95% CI） | *P* |  | n | AIP^c^ (95% CI） | *P* |  | n | AIP^c^ (95% CI） | *P* |
| Gender | Male | 11,628 | 1.00 |  |  | 816 | 1.00 |  |  | 1,621 | 1.00 |  |  | 9,191 | 1.00 |  |
|  | Female | 12,431 | 0.92 (0.86-1.00) | 0.038 |  | 751 | 0.90 (0.79-1.03) | 0.113 |  | 1,448 | 0.95 (0.85-1.06) | 0.371 |  | 10,232 | 0.92 (0.81-1.05) | 0.239 |
| Age^d^ (per 1 SD increment) | |  | 2.36 (2.25-2.47) | <0.001 |  |  | 1.99 (1.84-2.15) | <0.001 |  |  | 2.00 (1.86-2.14) | <0.001 |  |  | 2.84 (2.61-3.10) | <0.001 |
| Population size^e^ |  |  | 1.02 (0.98-1.06) | 0.288 |  |  | 1.01 (0.95-1.07) | 0.767 |  |  | 0.98 (0.93-1.04) | 0.530 |  |  | 1.07 (1.01-1.14) | 0.026 |
| No. of risk factors for aggravation^f^ | |  | 1.35 (1.28-1.43) | <0.001 |  |  | 1.13 (1.02-1.25) | 0.019 |  |  | 1.23 (1.12-1.34) | <0.001 |  |  | 1.59 (1.45-1.75) | <0.001 |
| No. of symptoms at diagnosis^g^ | | 19,423 | 1.16 (1.11-1.20) | <0.001 |  |  | 1.24 (1.16-1.32) | <0.001 |  |  | 1.20 (1.13-1.27) | <0.001 |  |  | 1.11 (1.03-1.20) | 0.006 |
| Variant type | Alpha | 1,567 | 1.00 |  |  |  |  |  |  |  |  |  |  |  |  |  |
|  | Delta | 3,069 | 1.36 (1.23-1.50) | <0.001 |  |  |  |  |  |  |  |  |  |  |  |  |
|  | Omicron | 19,423 | 0.23 (0.20-0.26) | <0.001 |  |  |  |  |  |  |  |  |  |  |  |  |
| Vaccination status^h^ | Unvaccinated | 7,239 | 1.00 |  |  | 1,540 |  |  |  | 2,592 | 1.00 |  |  | 3,107 | 1.00 |  |
|  | Dose 1 | 261 | 0.76 (0.61-0.93) | 0.009 |  | 4 |  |  |  | 128 | 0.85 (0.68-1.06) | 0.141 |  | 129 | 0.84 (0.45-1.58) | 0.587 |
|  | Dose 2 | 13,943 | 0.60 (0.53-0.68) | <0.001 |  | 1 |  |  |  | 236 | 0.75 (0.63-0.89) | 0.001 |  | 13,706 | 0.55 (0.46-0.66) | <0.001 |
|  | Dose 3 | 993 | 0.49 (0.37-0.66) | <0.001 |  | 0 |  |  |  |  |  |  |  | 993 | 0.40 (0.29-0.54) | <0.001 |
|  | Unknown | 1,623 | 0.98 (0.83-1.17) | 0.858 |  | 22 |  |  |  | 113 | 0.85 (0.62-1.18) | 0.335 |  | 1,488 | 0.84 (0.65-1.07) | 0.164 |

**Additional file 7C** AIP for extension of recovery period by SARS-CoV-2 variant type: COVID-19 patients not requiring hospitalization

|  |  | Entire period (n = 19,603) | | |  | SARS-CoV-2 variant type | | | | | | | | | | |
| --- | --- | --- | --- | --- | --- | --- | --- | --- | --- | --- | --- | --- | --- | --- | --- | --- |
|  |  |  |  |  |  | Alpha (n = 1,075) | | |  | Delta (n = 2,247) | | |  | Omicron (n = 16,281) | | |
|  |  | n | AIP^a^ (95% CI） | *P* |  | n | AIP^b^ (95% CI） | *P* |  | n | AIP^c^ (95% CI） | *P* |  | n | AIP^c^ (95% CI） | *P* |
| Gender | Male | 9,439 | 1.00 |  |  | 563 | 1.00 |  |  | 1,173 | 1.00 |  |  | 7,703 | 1.00 |  |
|  | Female | 10,164 | 1.08 (1.02-1.15) | 0.015 |  | 512 | 1.00 (0.89-1.13) | 0.994 |  | 1,074 | 1.13 (1.01-1.27) | 0.036 |  | 8,578 | 1.09 (0.99-1.20) | 0.069 |
| Age^d^ (per 1 SD increment) | |  | 1.19 (1.14-1.24) | <0.001 |  |  | 0.98 (0.90-1.06) | 0.564 |  |  | 1.15 (1.06-1.25) | 0.001 |  |  | 1.25 (1.18-1.32) | <0.001 |
| Population size^e^ |  |  | 0.99 (0.96-1.02) | 0.523 |  |  | 0.97 (0.92-1.02) | 0.259 |  |  | 1.06 (1.00-1.12) | 0.048 |  |  | 0.98 (0.93-1.02) | 0.290 |
| No. of risk factors for aggravation^f^ | |  | 1.03 (0.96-1.10) | 0.485 |  |  | 0.98 (0.82-1.16) | 0.770 |  |  | 1.04 (0.89-1.22) | 0.566 |  |  | 1.02 (0.94-1.11) | 0.668 |
| No. of symptoms at diagnosis^g^ | |  | 1.01 (0.97-1.04) | 0.615 |  |  | 1.02 (0.96-1.08) | 0.576 |  |  | 0.96 (0.90-1.02) | 0.145 |  |  | 1.05 (0.99-1.11) | 0.081 |
| Variant type | Alpha | 1,075 | 1.00 |  |  |  |  |  |  |  |  |  |  |  |  |  |
|  | Delta | 2,247 | 0.74 (0.68-0.81) | <0.001 |  |  |  |  |  |  |  |  |  |  |  |  |
|  | Omicron | 16,281 | 0.27 (0.24-0.30) | <0.001 |  |  |  |  |  |  |  |  |  |  |  |  |
| Vaccination status^h^ | Unvaccinated | 5,650 | 1.00 |  |  | 1,062 |  |  |  | 1,944 | 1.00 |  |  | 2,644 | 1.00 |  |
|  | Dose 1 | 190 | 0.80 (0.61-1.07) | 0.130 |  | 3 |  |  |  | 86 | 0.74 (0.53-1.04) | 0.079 |  | 101 | 0.98 (0.57-1.69) | 0.942 |
|  | Dose 2 | 11,881 | 0.63 (0.57-0.70) | <0.001 |  | 1 |  |  |  | 133 | 0.44 (0.31-0.63) | <0.001 |  | 11,747 | 0.69 (0.61-0.78) | <0.001 |
|  | Dose 3 | 762 | 0.38 (0.28-0.51) | <0.001 |  | 0 |  |  |  |  |  |  |  | 762 | 0.41 (0.30-0.55) | <0.001 |
|  | Unknown | 1,120 | 1.19 (1.04-1.37) | 0.013 |  | 9 |  |  |  | 84 | 0.45 (0.28-0.72) | 0.001 |  | 1,027 | 1.45 (1.22-1.72) | <0.001 |

AIP, adjusted incidence proportion; CI, confidence interval. ^a^Adjusted for covariates (i.e., gender, age, population size, No. of risk factors for aggravation, and No. of symptoms at diagnosis), vaccination status, and variant type. ^b^Adjusted for covariates. ^c^Adjusted for covariates and vaccination status.  ^d^Age was standardized (mean = 0; SD = 1). Age mean = 41.8 years; Age SD = 20.0 years. ^e^Population size was a discrete variable ranging from 1 to 4, where 1 represents the least populated (less than 30,000) and 4 represents the most populated (more than 120,000). ^f^Number of risk factors for aggravation was a discrete variable ranging from 0 to 2, with 2 representing 2 or more risk factors. ^g^Number of symptoms at diagnosis was a discrete variable ranging from 0 to 4, with 4 representing 4 or more symptoms. ^h^At least 14 days prior to infection.

**Additional file 8** Vaccination status ≥14 days after the first-dose and ≥7 days after the 2-3 doses

**8A** AIP for severe health consequences by SARS-CoV-2 variant type

|  |  | Entire period (n = 24,314) | | |  | SARS-CoV-2 variant type | | | | | | | | | |
| --- | --- | --- | --- | --- | --- | --- | --- | --- | --- | --- | --- | --- | --- | --- | --- |
|  |  |  |  |  |  | Alpha (n = 1,640) | | |  | Delta (n = 3,121) | | | Omicron (n = 19,553) | | |
|  |  | n | AIP^a^ (95% CI） | *P* |  | n | AIP^b^ (95% CI） | *P* |  | n | AIP^c^ (95% CI） | *P* | n | AIP^c^ (95% CI） | *P* |
| Gender | Male | 11,785 | 1.00 |  |  | 866 | 1.00 |  |  | 1,656 | 1.00 |  | 9,263 | 1.00 |  |
|  | Female | 12,529 | 0.55 (0.43-0.70) | <0.001 |  | 774 | 0.43 (0.27-0.67) | <0.001 |  | 1,465 | 0.58 (0.32-1.04) | 0.069 | 10,290 | 0.60 (0.42-0.85) | 0.004 |
| Age^d^ (per 1 SD increment) | |  | 4.00 (3.46-4.63) | <0.001 |  |  | 2.79 (2.21-3.54) | <0.001 |  |  | 3.40 (2.54-4.54) | <0.001 |  | 5.39 (4.21-6.91) | <0.001 |
| Population size^e^ |  |  | 0.97 (0.87-1.08) | 0.567 |  |  | 0.84 (0.68-1.04) | 0.102 |  |  | 1.26 (0.95-1.66) | 0.105 |  | 0.97 (0.84-1.12) | 0.686 |
| Number of risk factors for aggravation^f^ | |  | 1.54 (1.22-1.94) | 0.001 |  |  | 1.45 (1.05-2.01) | 0.023 |  |  | 1.42 (0.91-2.22) | 0.124 |  | 1.64 (1.19-2.27) | 0.005 |
| Number of symptoms at diagnosis^g^ | |  | 1.21 (1.06-1.39) | 0.004 |  |  | 1.23 (0.98-1.55) | 0.070 |  |  | 1.59 (1.19-2.14) | 0.002 |  | 1.10 (0.90-1.35) | 0.359 |
| Variant type | Alpha | 1,640 | 1.00 |  |  |  |  |  |  |  |  |  |  |  |  |
|  | Delta | 3,121 | 1.06 (0.73-1.53) | 0.768 |  |  |  |  |  |  |  |  |  |  |  |
|  | Omicron | 19,553 | 0.48 (0.33-0.69) | <0.001 |  |  |  |  |  |  |  |  |  |  |  |
| Vaccination status | Unvaccinated | 7,379 | 1.00 |  |  | 1,613 |  |  |  | 2,639 | 1.00 |  | 3,127 | 1.00 |  |
|  | Dose 1^h^ | 232 | 0.25 (0.07-0.95) | 0.042 |  | 4 |  |  |  | 111 | 0.19 (0.03-1.10) | 0.064 | 117 | 0.43 (0.05-3.43) | 0.428 |
|  | Dose 2^i^ | 13,754 | 0.23 (0.16-0.33) | <0.001 |  | 1 |  |  |  | 255 | 0.06 (0.01-0.41) | 0.004 | 13,498 | 0.29 (0.18-0.48) | <0.001 |
|  | Dose 3^i^ | 1,279 | 0.12 (0.05-0.29) | <0.001 |  | 0 |  |  |  |  |  |  | 1,279 | 0.12 (0.05-0.31) | <0.001 |
|  | Unknown | 1,670 | 0.95 (0.63-1.43) | 0.806 |  | 22 |  |  |  | 116 | 1.37 (0.41-4.59) | 0.608 | 1,532 | 0.96 (0.57-1.62) | 0.876 |

**8B** AIP for hospitalization by SARS-CoV-2 variant type

|  |  | Entire period (n = 24,059) | | |  | SARS-CoV-2 variant type | | | | | | | | | |
| --- | --- | --- | --- | --- | --- | --- | --- | --- | --- | --- | --- | --- | --- | --- | --- |
|  |  |  |  |  |  | Alpha (n = 1,567) | | |  | Delta (n = 3,069) | | | Omicron (n = 19,423) | | |
|  |  | n | AIP^a^ (95% CI） | *P* |  | n | AIP^b^ (95% CI） | *P* |  | n | AIP^c^ (95% CI） | *P* | n | AIP^c^ (95% CI） | *P* |
| Gender | Male | 11,628 | 1.00 |  |  | 816 | 1.00 |  |  | 1,621 | 1.00 |  | 9,191 | 1.00 |  |
|  | Female | 12,431 | 0.92 (0.86-1.00) | 0.036 |  | 751 | 0.90 (0.79-1.03) | 0.113 |  | 1,448 | 0.95 (0.85-1.06) | 0.369 | 10,232 | 0.92 (0.81-1.05) | 0.221 |
| Age^d^ (per 1 SD increment) | |  | 2.36 (2.25-2.48) | <0.001 |  |  | 1.99 (1.84-2.15) | <0.001 |  |  | 1.98 (1.85-2.13) | <0.001 |  | 2.87 (2.63-3.13) | <0.001 |
| Population size^e^ |  |  | 1.02 (0.99-1.06) | 0.274 |  |  | 1.01 (0.95-1.07) | 0.767 |  |  | 0.98 (0.93-1.04) | 0.538 |  | 1.07 (1.01-1.14) | 0.021 |
| Number of risk factors for aggravation^f^ | |  | 1.35 (1.28-1.43) | <0.001 |  |  | 1.13 (1.02-1.25) | 0.019 |  |  | 1.23 (1.12-1.35) | <0.001 |  | 1.59 (1.45-1.75) | <0.001 |
| Number of symptoms at diagnosis^g^ | |  | 1.16 (1.11-1.20) | <0.001 |  |  | 1.24 (1.16-1.32) | <0.001 |  |  | 1.20 (1.13-1.27) | <0.001 |  | 1.11 (1.03-1.20) | 0.009 |
| Variant type | Alpha | 1,567 | 1.00 |  |  |  |  |  |  |  |  |  |  |  |  |
|  | Delta | 3,069 | 1.36 (1.23-1.51) | <0.001 |  |  |  |  |  |  |  |  |  |  |  |
|  | Omicron | 19,423 | 0.23 (0.20-0.26) | <0.001 |  |  |  |  |  |  |  |  |  |  |  |
| Vaccination status | Unvaccinated | 7,239 | 1.00 |  |  | 1,540 |  |  |  | 2,592 | 1.00 |  | 3,107 | 1.00 |  |
|  | Dose 1^h^ | 230 | 0.70 (0.56-0.89) | 0.003 |  | 4 |  |  |  | 110 | 0.75 (0.58-0.96) | 0.022 | 116 | 0.88 (0.47-1.65) | 0.688 |
|  | Dose 2^i^ | 13,694 | 0.62 (0.55-0.69) | <0.001 |  | 1 |  |  |  | 254 | 0.79 (0.67-0.94) | 0.006 | 13,439 | 0.56 (0.47-0.68) | <0.001 |
|  | Dose 3^i^ | 1,273 | 0.49 (0.38-0.63) | <0.001 |  | 0 |  |  |  |  |  |  | 1,273 | 0.38 (0.28-0.50) | <0.001 |
|  | Unknown | 1,623 | 0.99 (0.83-1.17) | 0.891 |  | 22 |  |  |  | 113 | 0.86 (0.62-1.18) | 0.342 | 1,488 | 0.83 (0.65-1.07) | 0.143 |

**8C** AIP for extension of recovery period by SARS-CoV-2 variant type

|  |  | Entire period (n = 19,603) | | |  | SARS-CoV-2 variant type | | | | | | | | | |
| --- | --- | --- | --- | --- | --- | --- | --- | --- | --- | --- | --- | --- | --- | --- | --- |
|  |  |  |  |  |  | Alpha (n = 1,075) | | |  | Delta (n = 2,247) | | | Omicron (n = 16,281) | | |
|  |  | n | AIP^a^ (95% CI） | *P* |  | n | AIP^b^ (95% CI） | *P* |  | n | AIP^c^ (95% CI） | *P* | n | AIP^c^ (95% CI） | *P* |
| Gender | Male | 9,439 | 1.00 |  |  | 563 | 1.00 |  |  | 1,173 | 1.00 |  | 7,703 | 1.00 |  |
|  | Female | 10,164 | 1.08 (1.01-1.15) | 0.016 |  | 512 | 1.00 (0.89-1.13) | 0.994 |  | 1,074 | 1.13 (1.01-1.26) | 0.038 | 8,578 | 1.09 (0.99-1.19) | 0.073 |
| Age^d^ (per 1 SD increment) | |  | 1.19 (1.15-1.25) | <0.001 |  |  | 0.98 (0.90-1.06) | 0.564 |  |  | 1.15 (1.05-1.25) | 0.002 |  | 1.26 (1.19-1.33) | <0.001 |
| Population size^e^ |  |  | 0.99 (0.96-1.02) | 0.530 |  |  | 0.97 (0.92-1.02) | 0.259 |  |  | 1.06 (1.00-1.12) | 0.049 |  | 0.98 (0.93-1.02) | 0.304 |
| Number of risk factors for aggravation^f^ | |  | 1.03 (0.96-1.10) | 0.472 |  |  | 0.98 (0.82-1.16) | 0.770 |  |  | 1.04 (0.89-1.22) | 0.564 |  | 1.02 (0.94-1.11) | 0.648 |
| Number of symptoms at diagnosis^g^ | |  | 1.01 (0.97-1.04) | 0.688 |  |  | 1.02 (0.96-1.08) | 0.576 |  |  | 0.96 (0.90-1.01) | 0.137 |  | 1.05 (0.99-1.11) | 0.104 |
| Variant type | Alpha | 1,075 | 1.00 |  |  |  |  |  |  |  |  |  |  |  |  |
|  | Delta | 2,247 | 0.75 (0.69-0.81) | <0.001 |  |  |  |  |  |  |  |  |  |  |  |
|  | Omicron | 16,281 | 0.27 (0.24-0.30) | <0.001 |  |  |  |  |  |  |  |  |  |  |  |
| Vaccination status | Unvaccinated | 5,650 | 1.00 |  |  | 1,062 |  |  |  | 1,944 | 1.00 |  | 2,644 | 1.00 |  |
|  | Dose 1^h^ | 172 | 0.77 (0.57-1.04) | 0.083 |  | 3 |  |  |  | 78 | 0.72 (0.50-1.03) | 0.070 | 91 | 0.90 (0.50-1.64) | 0.737 |
|  | Dose 2^i^ | 11,680 | 0.64 (0.58-0.71) | <0.001 |  | 1 |  |  |  | 141 | 0.47 (0.33-0.66) | <0.001 | 11,538 | 0.69 (0.61-0.78) | <0.001 |
|  | Dose 3^i^ | 981 | 0.40 (0.31-0.52) | <0.001 |  | 0 |  |  |  |  |  |  | 981 | 0.42 (0.32-0.55) | <0.001 |
|  | Unknown | 1,120 | 1.19 (1.04-1.37) | 0.013 |  | 9 |  |  |  | 84 | 0.45 (0.28-0.72) | 0.001 | 1,027 | 1.44 (1.22-1.71) | <0.001 |

AIP, adjusted incidence proportion; CI, confidence interval. ^a^Adjusted for covariates (i.e., gender, age, population size, No. of risk factors for aggravation, and No. of symptoms at diagnosis), vaccination status, and variant type. ^b^Adjusted for covariates. ^c^Adjusted for covariates and vaccination status.  ^d^Age was standardized (mean = 0; SD = 1). Age mean = 41.8 years; Age SD = 20.0 years. ^e^Population size was a discrete variable ranging from 1 to 4, where 1 represents the least populated (less than 30,000) and 4 represents the most populated (more than 120,000). ^f^Number of risk factors for aggravation was a discrete variable ranging from 0 to 2, with 2 representing 2 or more risk factors. ^g^Number of symptoms at diagnosis was a discrete variable ranging from 0 to 4, with 4 representing 4 or more symptoms. ^h^At least 14 days prior to infection. ^i^At least 7 days prior to infection.
